# Bovine Derived *in vitro* Cultures Generate Heterogeneous Populations of Antigen Presenting Cells

**DOI:** 10.3389/fimmu.2019.00612

**Published:** 2019-03-29

**Authors:** Efrain Guzman, Myriam Pujol, Paolo Ribeca, Maria Montoya

**Affiliations:** ^1^The Pirbright Institute, Woking, United Kingdom; ^2^Doctoral Program in Agronomy Forestry and Veterinary Sciences, Universidad de Chile, Santiago, Chile; ^3^Centro de Investigaciones Biológicas (CIB - CSIC), Madrid, Spain

**Keywords:** bovine dendritic cells, bovine monocytes, bovine macrophages, bovine antigen presenting cells, *in vitro*-derived antigen presenting cells

## Abstract

Antigen presenting cells (APC) of the mononuclear phagocytic system include dendritic cells (DCs) and macrophages (Macs) which are essential mediators of innate and adaptive immune responses. Many of the biological functions attributed to these cell subsets have been elucidated using models that utilize *in vitro*-matured cells derived from common progenitors. However, it has recently been shown that monocyte culture systems generate heterogeneous populations of cells, DCs, and Macs. In light of these findings, we analyzed the most commonly used bovine *in vitro*-derived APC models and compared them to *bona fide* DCs. Here, we show that bovine monocyte-derived DCs and Macs can be differentiated on the basis of CD11c and MHC class II (MHCII) expression and that *in vitro* conditions generate a heterologous group of both DCs and Macs with defined and specific biological activities. In addition, skin-migrating macrophages present in the bovine afferent lymph were identified and phenotyped for the first time. RNA sequencing analyses showed that these monophagocytic cells have distinct transcriptomic profiles similar to those described in other species. These results have important implications for the interpretation of data obtained using *in vitro* systems.

## Introduction

Mononuclear phagocytic cells (MPC) are an important group of professional antigen presenting cells comprised mainly of monocytes, macrophages (Macs), and dendritic cells (DCs). These cells share a number of surface markers and are capable of phagocytosis, antigen presentation and immune regulation; however a number of factors that regulate differentiation, homeostasis and function of Macs and DCs remain largely unknown.

Over 20 years ago, human monocytes were shown to express CD14 on their surface exhibiting the capacity to differentiate into Macs or DCs in the presence of specific stimulating cytokines (1, 2). Since then, most studies of the human mononuclear phagocytic system have used *in vitro*-generated monocyte-derived macrophages (MoMacs) and monocyte-derived DCs (MoDCs) mainly due to the scarcity of these cells in peripheral blood (3, 4). Even though monocytes-macrophages and DCs belong to two different lineages (5), when entering tissues, monocytes can differentiate into cells that share morphological and functional features with either dendritic cells (DCs) or Macs. MoDCs have been observed at mucosal tissues and in inflammatory settings where they are usually referred to as “inflammatory DCs” (6).

Bovine blood monocytes express CD14 on their surface just like their human and mouse orthologs. Bovine MoDCs were shown to differentiate from monocyte progenitors by culturing these cells with granulocyte-macrophage colony stimulating factor (GM-CSF) and interleukin 4 (IL-4), with or without addition of Flt3 ligand (Flt3L) and the resulting cells were shown to be non-adherent (7), maintained their expression of CD14 whereas CD1b expression increased compared to their monocyte progenitors. In contrast, Seo et al. (8) generated bovine MoDCs with staphylococcal enterotoxin C1 and these cells were adherent, lost their CD14 expression and were phenotyped as CD68^−^, CD163^−^, DC-SIGN^+^, MHCII^high^, CD11a^low^, CD11b^high^, CD11c^high^, and CD1b^high^. *Bona fide* bovine DCs obtained by cannulation of lymphatic vessels have been identified as being large in size (FSC^high^) expressing high levels of CD205 and MHCII (9).

Various methods have also been used to generate bovine MoMacs: Abdellrazeq et al. (10) and Magee et al. (11) cultured blood monocytes in plastic plates in the absence of any cytokines and defined MoMacs as adherent cells; Werling et al. (12) cultured MoMacs in Teflon bags in the presence of an amino acid rich medium and others generated MoMacs using GM-CSF (7, 13). The resulting cells were heterogeneous with adherent and non-adherent populations. Nevertheless, in most of the examples mentioned above the resulting cells were treated as single homogeneous populations.

Recent advances in gene transcription profiling and an increased availability of immunological reagents have permitted a thorough and comparative characterization of the various members of the MPC system (14, 15) across different species. These reagents, techniques and approaches are constantly being reviewed and the data generated updated. The review by Guilliams et al. (5) provides a conceptual framework for interpreting the extensive information available on MPC from studies in humans and mice. In addition, Auray et al. (14) have attempted to summarize what is known about MPC in other mammalian species and have highlighted the various gaps in knowledge, including the differences and similarities between different species. They suggested to use the same terminology for humans and mice in the study of MPC subsets carrying out the same functional activity in other species. In the specific case of the bovine system, Park et al. (16) and more recently Talker et al. (17) took advantage of larger blood supply available in cattle to phenotype blood DC.

The notion that *in vitro*-derived DCs and Macs were composed of homogeneous populations of cells was challenged by Helft et al. (18). Using the mouse system, they have shown that bone marrow (BM)-derived DCs and Macs cultures are in fact a heterogeneous population of both, DCs and Macs differentially expressing MHCII and CD11c and exclusively expressing Zbtb46 and MerTK, respectively.

Taking advantage of the availability of *bona fide* and uncultured DCs obtained from the pseudo-afferent lymph, which are not normally available in mouse or human studies, we have used the approach described by Helft et al. to analyse *in vitro* models of bovine DCs and compared them to *bona fide* DCs. Firstly, we confirmed previous data (19–21) describing *bona fide* afferent lymph DCs (ALDCs) as MHCII^++^CD11c^+^CD11b^−/+^CD205^+^CD1b^+/++^CD14^−^CD172a^++/+^ and during this process bovine macrophages in afferent lymph (ALMacs) were identified for the first time. These ALMacs were defined as MHCII^+^CD11c^+^CD11b^+^CD205^−^CD1b^+^CD14^−^CD172a^+^ and comprise about 10% of the total number of cells in the bovine afferent lymph. Secondly, our results showed that monocyte-derived *in vitro* cultures of MPC are comprised of both DCs and Macs where DCs/Macs ratio varies depending on many factors. According to ALDCs and ALMacs phenotype, MoDCs can be defined as: MHCII^++^CD11c^+^CD11b^+^CD205^+^CD1b^+/++^CD14^+^CD172a^−^ whereas MoMacs can be defined as: MHCII^+^CD11c^+^CD11b^+^CD205^−^CD1b^+^CD14^+^CD172a^+^. Our approach was comprised of three phases: firstly, identification of distinct subsets of bovine MPC by flow cytometry, microscopy and functional assays; secondly, an unbiased classification based on RNA-sequencing; thirdly, validation of transcriptomic data by evaluating transcription of a selection of genes that were identified. These findings will prove valuable for further studies focused on characterizing the function of the individual subsets of DC and Macs.

## Materials and Methods

### Bovine Cells

Heparinized peripheral blood was obtained from six conventionally reared, MHC-defined *Bos taurus* (Holstein-Friesian cattle) by venepuncture of a superficial venous vessel. Details of cattle MHC haplotypes, alleles, and nomenclature can be found at http://www.ebi.ac.uk/ipd/mhc/bola/. Heparinized venous blood was centrifuged for 30 min at 300 × g over Histopaque 1083 (Sigma-Aldrich) and the mononuclear cells (PBMC) were washed three times in phosphate buffered saline (PBS). Bovine CD14^+^ cells were purified by magnetic antibody cell sorting (MACS) using anti-human CD14^+^ microbeads (Miltenyi Biotec), shown to bind the bovine ortholog (22), following the manufacturer's instructions. To prepare antigen-presenting cells (APCs), 1 × 10^6^ CD14^+^ were incubated in 6 well plates (Nunc) for 7 days at 37°C in 3 ml of RPMI 1640 medium (Invitrogen) containing 10% heat-inactivated FCS, 2 mM L-glutamine, 55 μM 2-mercaptoethanol and 1% penicillin/streptomycin (Sigma-Aldrich) and supplemented with recombinant bovine GM-CSF with or without recombinant bovine IL4 and human rFlt-3L (100 ng/ml; Genzyme, West Malling, Kent, UK) (13, 23–25).

Pseudo-afferent lymph cells were obtained by cannulation of lymphatic vessels of six MHC-defined Holstein-Friesian calves as described before (9, 19). The mononuclear cells were isolated from the afferent lymph by density gradient centrifugation over Histopaque 1086 (Sigma) as described above.

Alveolar macrophages (AlvMacs) were obtained as described (26). Briefly, lungs were lavaged with PBS containing 1% penicillin/streptomycin and 1 μg/ml of fungizone (Sigma-Aldrich) at post-mortem. Mononuclear cells were separated by density centrifugation over Histopaque 1083 as described above. AlvMacs were defined as MHCII^+^CD11c^+^CD11b^+^CD205^−^CD1b^+^CD14^−^CD172a^+^ and the cells obtained were over 92% pure as defined by flow cytometry.

All regulated procedures were carried out according to the UK's Animals (Scientific Procedures) Act 1986 and following ARRIVE guidelines. All work was approved by the Pirbright Institute's local ethics committee.

### Monoclonal Antibodies and Flow Cytometry

Most antibodies used in these studies have been described before. Fluorochrome-labeled mouse anti-bovine monoclonal antibodies (mAbs) obtained from The Pirbright Institute were: CC98 (anti-CD205, IgG2b), CC14 (anti-CD1b, IgG1), CC149 (anti-SIRPα/CD172a, IgG2b), CC126 (anti-CD11b, IgG2b), ILA-16 (anti-bovine CD11c, IgG1), IL-A21 (anti-MHC II, IgG2a), ILA-156 (anti-CD40, IgG1), N32/52-3 (anti-CD80, IgG1), ILA-159 (anti-CD86, IgG1). 209MD26A (anti-bovine CD209/DC-SIGN, IgG2a) was obtained from Kingfisher Biotech. Anti-human mAbs KD1 (anti-CD16, IgG2a, Serotec Bio-Rad) and TuK4 (anti-CD14 VioGreen, IgG2a, Miltenyi Biotec) have been shown to cross-react with their bovine orthologs (7, 9, 19, 22, 23). Affinity-purified, fluorochrome-conjugated, isotype- and concentration matched monoclonal antibodies (The Pirbright Institute) were used as controls: TRT1 (IgG1) and TRT3 (IgG2a) raised against turkey rhinotracheitis virus (27) and AV29 (IgG2b) raised against avian CD4 (28). Dead cells were excluded using the Live/Dead nIR Fixable Staining kit (Thermo) following the manufacturer's instructions.

Antibodies obtained from The Pirbright Institute were purified over protein A/G columns (Pierce). All antibodies were conjugated to various fluorochromes using Lightning-Link kits (Innova Biosciences) following the manufacturer's instructions or obtained directly conjugated from the manufacturer where indicated. All monoclonal antibodies were titrated to determine optimal use concentration. For staining, cells were washed in PBS and resuspended in PBS containing the appropriate fluorochrome-conjugated antibodies (1 μg/10^6^ cells) and Live/Dead nIR viability dye (0.5 μl/10^6^ cells). After a 1 h incubation at 4°C in the dark, the cells were washed three times with PBS and fixed using Cytofix/Perm buffer (BD Biosciences).

Quantitative flow cytometry using Quantibrite beads (BD) was used to calculate the number of absolute molecules of the cell's surface using a 1:1 ratio of beads to PE:mAb conjugate using the manufacturer's instructions. A linear regression of Log_10_ PE molecules/bead was used to generate a standard curve (GraphPad Prism v7) and to determine the number of antigen molecules/cell.

For each experiment, a minimum of 50,000 live/single events was recorded using a BD LSRFortessa cytometer). Compensations were automatically calculated using single-stained controls and isotype controls using BD's automatic compensation tool. Cytometry analysis was performed using FlowJo vX for PC (TreeStar).

### Phagocytosis

The ability of cell subsets to phagocytose bioparticles was determined using the pHrodo Red *E. coli* BioParticles Conjugate kit (ThermoFisher) following the manufacturer's instructions. Mixed cultures of Mo-derived cells (generated by incubating CD14^+^ MACS-sorted blood monocytes with rboGM-CSF, rbo-IL4 and rFlt3L as described above) or AL-derived cells (obtained *ex vivo* as described above) were incubated with pHrodo BioParticles at 37 or 4°C in tissue culture media for 15 min after which the cells were washed with the kit's buffers following the manufacturer's instructions. Cells were then surfaced stained as described above with anti-MHCII, -CD11c, and -CD205 antibodies and analyzed by flow cytometry. Cell subsets in the mixed cultures were defined post-acquisition as follows: MoMacs (MHCII^+^CD11c^+^CD205^−^), MoDCs (MHCII^++^CD11c^+^CD205^+^), ALMacs (MHCII^+^CD11c^+^CD205^−^), and ALDCs (MHCII^++^CD11c^+^CD205^+^).

### Cell Sorting

Cells subsets were stained as described above, passed through a 70 μm filter mesh (Fisher) and flow sorted using a FACSAria IIIU into 15 ml Falcon tubes containing either FCS or RNAlater. An example of the gating strategy and resulting sorted populations is shown in Supplementary Figure 1. A minimum of 100,000 live/single events was collected.

### mRNA Sequencing

The following sorted populations (as described above) were used for mRNA sequencing: (1) blood monocytes (*ex vivo* CD14^+^); (2) alveolar macrophages (MHCII^+^, CD11c^+^ CD205^−^); (3) MoMacs (MHCII^+^, CD11c^+^, CD205^−^); (4) MoDCs (MHCII^++^, CD11c^+^, CD205^+^); (5) ALMacs (MHCII^+^, CD11c^+^, CD205^−^); (6) ALDCs (MHCII^++^, CD11c^+^, CD205^+^). A minimum of 100,000 live/single cells was collected. Purity of the resulting populations was >95% as determined by flow cytometry post-sort. Total RNA from flow sorted cell subsets was obtained using Qiagen's Total RNA kit following the manufacturer's instructions. RNA QC was performed on a Bioanalyzer 2100 using the RNA pico chip. Samples were selected based on the sequencing kit manufacturer's requirements for RNA integrity (RNAi, >7). Sequencing libraries were prepared from total RNA using the SMART-Seq v4 Ultra Low Input RNA Kit (Takara-Clontech, Saint-Germain-en-Laye, France) in combination with a Nextera XT Library preparation kit (Illumina, Chesterford UK) according to the manufacturer's instructions. Samples were individually indexed for pooling using a dual index strategy. Libraries were quantified on a Tapestation DNA 1000 Screen tape (Agilent, Cheadle UK) and by qPCR using an NGS Library Quantification Kit (KAPA Biosystems, London UK) on an AriaMx qPCR system (Agilent). Libraries were then normalized, pooled, diluted and denatured for sequencing on the NextSeq 500 (Illumina) according to the manufacturer's instructions. Samples were pooled such that a minimum of 10M unique clusters per sample was achieved. PhiX control library (Illumina) was spiked into the main library pool at 5% v/v for quality control and library diversity balancing purposes. Sequencing was performed using a mid-output flow cell with 2 × 75 cycles of sequencing providing 260M reads. RNA-Seq studies were carried out by Cambridge Genomic Services, Cambridge UK.

The high-throughput RNA sequencing data were subjected to preliminary quality control, and then processed with a computational pipeline for primary data analysis based on the GEM mapper (29), which is an evolution of the one used to process the data produced by the GEUVADIS consortium (30). The pipeline is robust and adequate for the analysis of data obtained from non-model species—for instance it includes a highly sensitive *de-novo* intron discovery step, to compensate for errors or limitations in the available annotation of cellular transcript. Read counts for transcripts were obtained for all samples from the results of the pipeline.

### Quantitative RT-PCR

Total RNA extracted for high throughput sequencing or extracted from freshly isolated cells using the RNeasy Plus Kit (Qiagen) was used to determine gene-specific transcription by qRT-PCR. RNA was quantified using a NanoDrop (Thermo Fisher), reverse transcribed and amplified using the SuperScript III qRT-PCR kit (Life Sciences) following the manufacturer's instructions. Gene-specific primers and probes (Supplementary Table 1) were designed and validated using OligoArchitect (Sigma) and synthesized commercially (Sigma). qRT-PCR reactions were carried out using a QuantStudio 5 (ThermoFisher) as follows: 50°C for 15 min, 95°C for 2 min, followed by 40 cycles of 95°C for 15 s, and 60°C for 30 s. Calibration curves were performed and a primer matrix was carried out to optimize the concentration of each primer. All data were standardized to the endogenous control, 18S ribosomal RNA (18S rRNA) and analyzed as described before (31).

### Statistics

The data described for each figure were analyzed using one-way analysis of variance (ANOVA) and pairwise comparison using GraphPad Prism v7 and expressed as means ± standard deviation (SD). The normality of data distribution was determined using the Kolmogorov-Smirnov test. The differences between groups were determined using the Mann-Whitney or one-way ANOVA test and differences within each group were determined using the Dunn's or Tukey *post-hoc* test. Statistical significance was assumed when p-value was ^*^p < 0.05, ^**^p < 0.01, and ^***^p < 0.001.

Statistical analyses of differential gene expression from high-throughput sequencing data were performed as follows: first the matrix of counts per transcript and condition was processed with edgeR (Bioconductor). A relevant subset of the list of differentially expressed genes thus obtained was selected by imposing suitable thresholds on Fold-Change [log_2_ (FC) ≥ 1] and False Discovery Rate (FDR ≤ 0.05) (**Figure 4C**). Second, we sought to characterize the samples by clustering them into groups having similar gene regulation according to their RNA expression. To do so, we took as a starting point the Multi-Dimensional Scaling (MDS) analysis offered by edgeR (**Figure 4A**); it considers as input a matrix containing for each couple of samples the RMS of the log fold-change of the 500 transcripts that change most between the two conditions, and it projects it onto a 2-dimensional space. While this technique is usually able to effectively reveal the relations between samples, it cannot be used to understand which transcripts are responsible for the different behaviors of the clusters. However, as it often happens, correspondence analysis (CA, **Figure 4B**), which is particularly suited to categorical and compositional data, produces a clustering entirely similar to that of MDS; in addition, CA does allow the variation between clusters to be decomposed and attributed to single transcripts (Table 3). CA is an unsupervised learning technique that generalizes Principal Component Analysis—we did not use PCA on this occasion because the correspondence between MDS and PCA was not as good as the one between MDS and CA (data not shown).

## Results

### Bovine Monocyte-Derived Cultures Are Heterogeneous Populations

**Figure 1:**
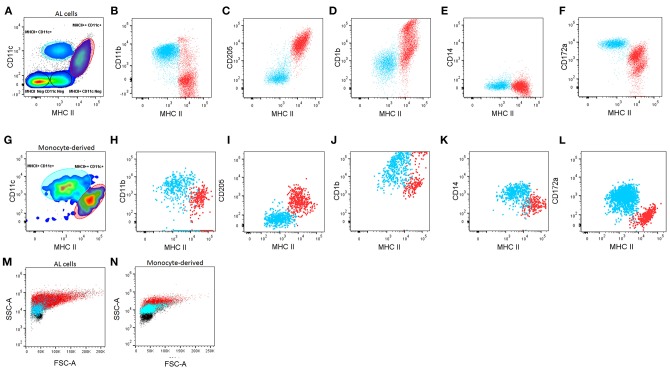
Characterization of macrophages (Macs) and dendritic cells (DCs) from the bovine afferent lymph (AL, **A–F**) and *in vitro*-derived from blood monocytes (Mo, **G–L**). **(A, G)**. Based on the differential expression of CD11c and MHC class II, DCs and Macs can be defined in both, the AL and in Mo cultured *in vitro* with GM-CSF, IL-4, and Flt3L. DCs (red) and Macs (blue) can be further differentiated based on surface expression of CD11b **(B,H)**, CD205 **(C,I)**, CD1b **(D,J)** CD14 **(E,K**) and CD172a **(F,L)**. FSC/SSC plots of DCs (red) and Macs (blue) compared with other lymphocytes present in the cultures (black) **(M,N)**. A minimum of 50,000 single/live events were analyzed. Adherent and non-adherent cells were found to have similar phenotypes separately, therefore the mixture of both of these is shown. Figure representative of cells from 6 different animals analyzed in duplicate.

Previously, DCs present in the AL (ALDCs) were defined as FSC^high^CD205^+^CD14^−^, CD172^+/++^ (19), but ALMacs had not been identified. Using the approach by Helft et al (18), staining AL cells for expression of MHCII and CD11c reveals the presence of four distinct populations (Figure 1A): (1) MHCII^++^CD11c^+^; (2) MHCII^+^CD11c^+^; (3) MHCII^+^CD11c^−^; (4) MHCII^−^CD11c^−^. The latter two were identified as being B and T cells, respectively by the differential expression of sIg and CD3 respectively (data not shown). In addition, we identified the presence of SSC^high^ MHC^low^ CD14^int^ and a separate SSC^low^ MHC^low^ CD14^high^ population (data not shown), similar to those previously identified in sheep afferent lymph as granulocytes and monocytes, respectively (32).

The MHCII^++^CD11c^+^ and MHCII^+^CD11c^+^ populations were characterized further based on their expression of CD11b, CD205, CD1b, CD14, and CD172a (Figures 1B–F). Based on these results, ALDCs were identified as: MHCII^++^CD11c^+^CD11b^−/+^CD205^+^CD1b^+/++^CD14^−^ CD172a^++/+^. On the other hand, ALMacs were identified as: MHCII^+^CD11c^+^CD11b^+^CD205^−^CD1b^+^CD14^−^CD172a^+^. Therefore, ALDCs and ALMacs were defined by their differential expression of MHCII and CD205. Based on these phenotypes, ALDCs comprised 10% (±5%) of all cells in the afferent lymph; similarly, ALMacs comprised 19% (±4%) of all cells in the afferent lymph (Supplementary Figure 1).

Bovine blood monocytes phenotype has been extensively compared with the resulting MoDCs and MoMacs by one-dimensional immunophenotyping; MoDCs were originally characterized by their increased expression of the non-classical MHC molecule CD1b (7) and their non-adherent phenotype, whereas MoMacs have generally been recognized as being adherent (7, 13). Following the approach by Helft et al (18) and having identified DCs present in the AL, blood monocytes cultured in the presence of GM-CSF, IL4, and Flt3L where characterized. Both adherent and non-adherent cells were present in cultures and these two cell types were phenotyped separately (data not shown), however cytometric analysis revealed that both adherent and non-adherent cells comprised mixed populations of MHCII^++^CD11c^+^ and MHCII^+^CD11c^+^ (Figure 1G). According to the phenotype of ALDCs and ALMacs, MoDCs can be defined as: MHCII^++^CD11c^+^CD11b^+^CD205^+^CD1b^+/++^CD14^+^CD172a^−^ whereas MoMacs can be defined as: MHCII^+^CD11c^+^CD11b^+^CD205^−^CD1b^+^CD14^+^CD172a^+^ (Figures 1H–L). Due to the fact that both adherent and non-adherent cells contained mixed populations of DCs and Macs, all subsequent studies were carried out without separating adherent and non-adherent cells.

Previously, FSC/SSC plots were used in identifying differentiated DCs from their progenitors. FSC/SSC analysis shows that both ALDCs and MoDCs vary in size, from low FSC (~8 μm when compared to lymphocytes) to very high FSC. In contrast, macrophages have a more defined size, generally similar to that of lymphocytes. Macrophages appear to be more granular than lymphocytes and DCs more granular than macrophages (Figures 1M,N). Cytospins of flow-sorted, Giemsa-stained ALDCs and ALMacs confirmed their different physical characteristics (Supplementary Figure 2). Culturing bovine blood monocytes with GM-CSF and IL4 but without Flt3L, or without both IL4 and Flt3L did not result in the preferential growth of DCs over Macs; these cultures contained variable ratios of MoDCs/MoMacs and they never “polarized” one way or another. Therefore, phenotypic analysis confirmed that, similar to the mouse system, bovine monocyte-derived cultures were comprised of mixed populations which can be differentiated based on their differential expression of MHCII, CD11c and CD205.

In light of these results and for all subsequent studies, blood monocytes were cultured with GM-CSF, IL4, and Flt3L; both adherent and non-adherent cells were mixed and the resulting cells were defined simply as follows: blood monocytes (*ex vivo* CD14^+^), MoMacs (MHCII^+^CD11c^+^CD205^−^), MoDCs (MHCII^++^CD11c^+^CD205^+^), ALMacs (MHCII^+^CD11c^+^CD205^−^), and ALDCs (MHCII^++^CD11c^+^ CD205^+^).

### Blood Monocytes, Macs, and DCs Express Different Amounts of Co-stimulatory Molecules on Their Surface

**Figure 2:**
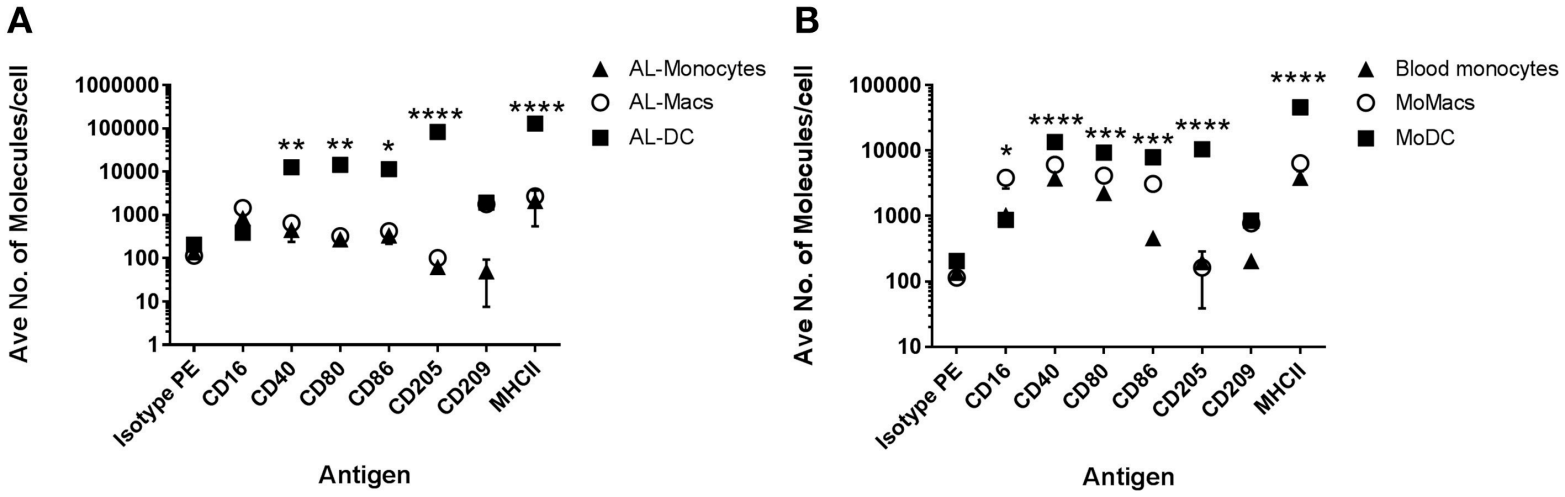
Quantification of surface molecules on bovine AL monocytes, ALDCs, ALMacs, peripheral blood monocytes, MoMacs, MoDCs. **(A)** Quantification of the average number of molecules on the surface of bovine AL monocytes (black triangles), ALMacs (white circles) and ALDCs (black squares). **(B)** Average number of molecules on the surface of bovine blood monocytes (black triangles), MoMacs (white circles), and MoDCs (black squares). Each symbol represents means calculated from cells obtained from 5 different animals analyzed in triplicate; error bars indicate standard error of the means. *p < 0.05, **p < 0.01, *** *p* < 0.001, and ****p < 0.0001.

One of the principal functional differences between DCs and Macs is their differential capacity to stimulate T cells. Expression of co-stimulatory molecules by flow cytometry has typically been performed on the basis of mean fluorescence intensity (MFI) as a proxy measure of antigen expression. A regression analysis of fluorescence in relation to antigen expression was performed to determine the absolute number of molecules on bovine blood and AL-derived monocytes, DCs and Macs cell's surface. AL monocytes and ALMacs had comparable number of all antigens on their surface except for CD209, where ALMacs had more CD209 molecules than AL monocytes, however this difference was not statistically significant (Figure 2A and Table 1). ALDCs had significantly higher number of CD40, CD80, CD86, CD205, and MHCII, whereas both cell types had similar number of CD209 (DC-SIGN) molecules on their surface (Figure 2A and Table 1). When comparing blood monocyte-derived cells, MoDCs had significant higher number of CD40, CD80, CD86, CD205, and MHCII compared with MoMacs, whereas both cell types had similar number of CD209 (DC-SIGN) molecules on their surface (Figure 2B and Table 2). The expression levels of CD16 was similar in all AL-derived cells; however MoMacs expressed significantly higher number of CD16 molecules than MoDCs or blood monocytes (Figure 2B and Table 2).

**Table 1:** Statistical analysis of number of molecules on bovine AL monocytes, ALDC vs. ALMacs.

**Isotype PE**	**Isotype PE**	**Mean Diff**.	**Significance**	**Adjusted *P*-Value**
	AL-Macs vs. AL-DC	−91	ns	0.9996
	AL-Macs vs. AL-Monocytes	−21	ns	>0.9999
	AL-DC vs. AL-Monocytes	70	ns	0.9998
**CD16**				
	AL-Macs vs. AL-DC	1,070	ns	0.948
	AL-Macs vs. AL-Monocytes	605	ns	0.983
	AL-DC vs. AL-Monocytes	−465	ns	0.99
**CD40**				
	AL-Macs vs. AL-DC	−11,992	^**^	0.0051
	AL-Macs vs. AL-Monocytes	203	ns	0.9981
	AL-DC vs. AL-Monocytes	12,195	^**^	0.0044
**CD80**				
	AL-Macs vs. AL-DC	−14,100	^**^	0.0011
	AL-Macs vs. AL-Monocytes	50	ns	0.9999
	AL-DC vs. AL-Monocytes	14,150	^**^	0.0011
**CD86**				
	AL-Macs vs. AL-DC	−10,975	^*^	0.0104
	AL-Macs vs. AL-Monocytes	90	ns	0.9996
	AL-DC vs. AL-Monocytes	11,065	^**^	0.0098
**CD205**				
	AL-Macs vs. AL-DC	−82,399	^****^	<0.0001
	AL-Macs vs. AL-Monocytes	39	ns	>0.9999
	AL-DC vs. AL-Monocytes	82,438	^****^	<0.0001
**CD209**				
	AL-Macs vs. AL-DC	−152	ns	0.9989
	AL-Macs vs. AL-Monocytes	1,725	ns	0.8708
	AL-DC vs. AL-Monocytes	1,877	ns	0.849
**MHCII**				
	AL-Macs vs. AL-DC	−1,27,210	^****^	<0.0001
	AL-Macs vs. AL-Monocytes	592	ns	0.9838
	AL-DC vs. AL-Monocytes	1,27,802	^****^	<0.0001

* p < 0.05,

** p < 0.01,

*** p < 0.001, and

**** *p < 0.0001*.

**Table 2:** Statistical analysis of number of molecules on bovine blood monocytes vs. MoDCs vs. MoMacs.

**Isotype PE**		**Mean Diff**.	**Significance**	**Adjusted *P*-Value**
	MoDC vs. MoMacs	91	ns	0.9962
	Monocytes vs. MoMacs	21	ns	0.9998
	Monocytes vs. MoDC	−70	ns	0.9978
**CD16**				
	MoDC vs. MoMacs	−2,975	^*^	0.0323
	Monocytes vs. MoMacs	−2,825	^*^	0.0433
	Monocytes vs. MoDC	150	ns	0.9898
**CD40**				
	MoDC vs. MoMacs	7,400	^****^	<0.0001
	Monocytes vs. MoMacs	−2,350	ns	0.1042
	Monocytes vs. MoDC	−9,750	^****^	<0.0001
**CD80**				
	MoDC vs. MoMacs	5,150	^***^	0.0003
	Monocytes vs. MoMacs	−1,900	ns	0.2167
	Monocytes vs. MoDC	−7,050	^****^	<0.0001
**CD86**				
	MoDC vs. MoMacs	4,800	^***^	0.0006
	Monocytes vs. MoMacs	−2,640	ns	0.0617
	Monocytes vs. MoDC	−7,440	^****^	<0.0001
**CD205**				
	MoDC vs. MoMacs	10,338	^****^	<0.0001
	Monocytes vs. MoMacs	35	ns	0.9994
	Monocytes vs. MoDC	−10,303	^****^	<0.0001
**CD209**				
	MoDC vs. MoMacs	75.5	ns	0.9974
	Monocytes vs. MoMacs	−570	ns	0.8638
	Monocytes vs. MoDC	−645	ns	0.8291
**MHCII**				
	MoDC vs. MoMacs	39,483	^****^	<0.0001
	Monocytes vs. MoMacs	−2,562	ns	0.0713
	Monocytes vs. MoDC	−42,044	^****^	<0.0001

* p < 0.05,

** p < 0.01,

*** p < 0.001, and

**** *p < 0.0001*.

### Bovine MoDCs and MoMacs Have Differential Capacity to Phagocytose

**Figure 3:**
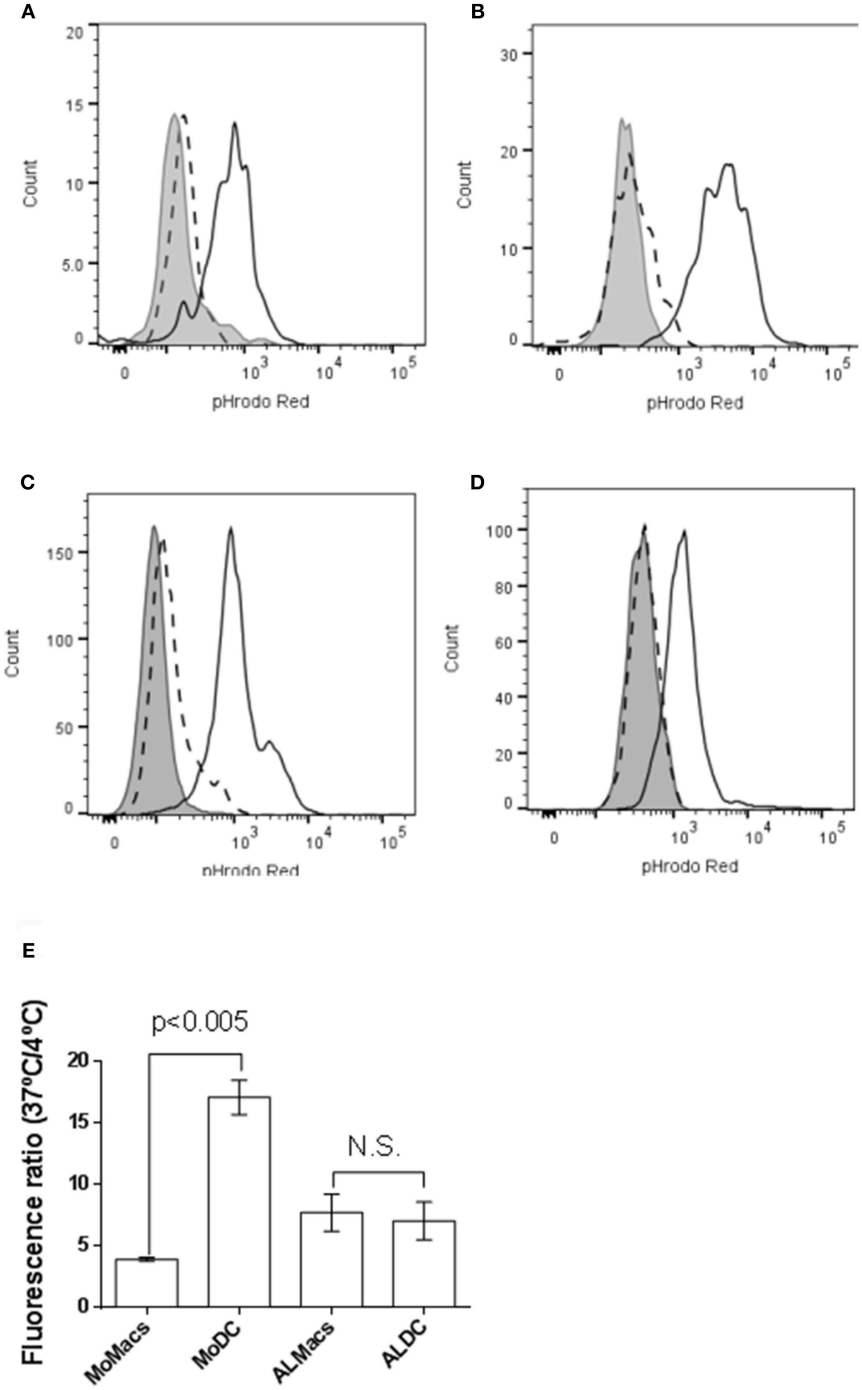
Phagocytosis activity of bovine afferent lymph and monocyte-derived cells. Phagocytic activity of MoMacs **(A)**, MoDCs **(B)**, ALMacs **(C)**, and ALDCs **(D)** was assessed using pHrodo Red *E. coli* BioParticles. Each histogram is representative of cells from 5 different animals. Gray histograms represent background fluorescence; dashed histograms represent cells incubated with pHrodo Red *E. coli* bioparticles at 4°C and solid white histograms represents fluorescence of cells incubated with bioparticles at 37°C. **(E)** Quantification of phagocytosis for each cell type. Bars represent the mean fluorescence intensity ratios (37°C/4°C) for cells from 5 different animals. Error bars indicate standard deviations.

Another functional difference between DCs and Macs is their capacity to phagocytose. It has previously been shown that human MoMacs have a better phagocytic capacity compared to human MoDCs (33). Therefore, the capacity of mixed populations of bovine Macs and DCs (cultured and defined as described above) to phagocytose fluorescent *E. coli* was investigated. It was decided not to flow-sort Macs and DCs prior to the phagocytosis assays to prevent undesired activation or cell death, consequently DCs and Macs were identified post-acquisition. In contrast to the data published before (34), bovine MoDCs had a higher capacity to phagocytose *E coli* particles than MoMacs (Figures 3A,B) as defined by the ratio of intracellular fluorescence at 4 and 37°C (Figure 3E). In contrast, there was no significant difference in the phagocytic capacity of ALMacs compared to ALDCs (Figures 3C–E). These results suggested that bovine MoMacs/MoDCs cultures with higher or lower phagocytic capacities were influenced by the MoMacs/MoDCs ratio in the culture itself. The conclusions drawn from these types of functional assays need to be assessed in the context of the heterogeneous nature of the cultures.

### Bovine DCs and Macs Have Distinct Global Transcription Signatures

Gene transcription profiles through the use of microarrays or now more commonly high throughput sequencing, have been used to define specific subsets of the MPC system in various systems (35). However, a comparison of global transcription profiles of DCs and Macs has not been performed in the bovine system. Using high throughput sequencing, global transcription profiles of flow-sorted bovine MoMacs (both adherent and non-adherent MHCII^+^, CD11c^+^, CD205^−^), MoDCs (both adherent and non-adherent MHCII^++^, CD11c^+^, CD205^+^), ALMacs (MHCII^+^, CD11c^+^, CD205^−^), and ALDCs (MHCII^++^, CD11c^+^, CD205^+^) were carried out. Blood monocytes (*ex vivo* CD14^+^) and alveolar macrophages (MHCII^+^, CD11c^+^, CD205^−^) were used as internal controls.

**Figure 4:**
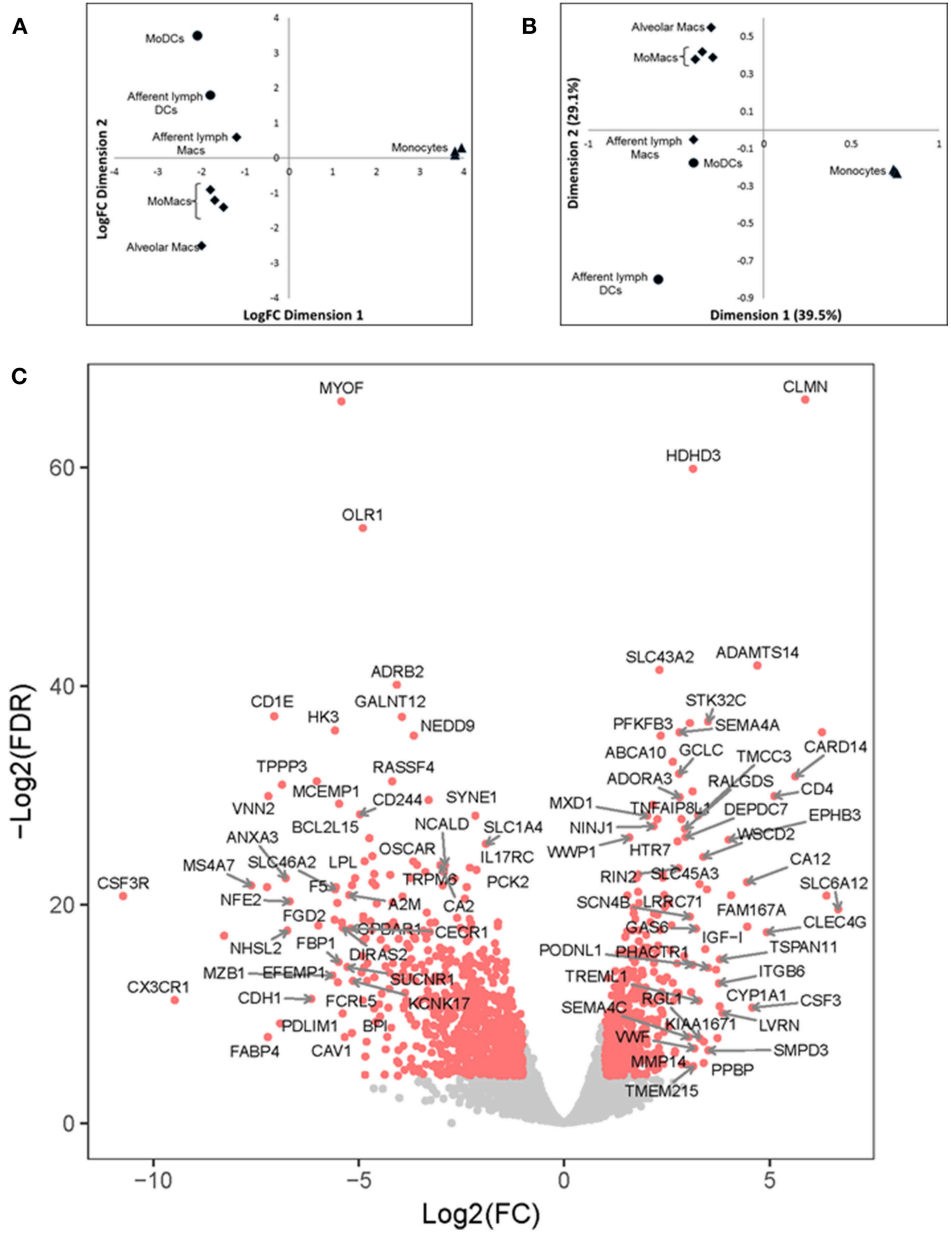
Differentially-expressed (DE) genes in bovine blood monocytes, Macs and DCs obtained from the afferent lymph and those obtained *in vitro* from monocyte progenitors. Bovine blood monocytes (CD14^+^, triangles), Macs (diamonds), and DCs (circles, *N* = 4, as defined in Figure 1) were flow sorted and total RNA extracted. RNA sequencing was performed on a NextSeq 500 system and DE analysis was performed as described and Materials and Methods. **(A)** Multi-Dimensional Scaling (MDS) analysis on leading log fold-change (500 most expressed genes) as performed by edgeR on samples. **(B)** Bi-plot of Correspondence Analysis (CA) on samples (no genes drawn). In both cases the circles represent DCs, diamonds represent Macs and triangles represent blood monocytes. **(C)** Volcano plot showing differential expression (LogFC) vs. statistical significance (LogFD) of grouped DCs vs. Macs. Red dots represent differentially expressed (*p* < 0.001). The 20 genes giving the largest contribution to inertia are listed in Table 3.

Multi-dimensional scaling (MDS) analysis was performed taking into account the 500 genes with the highest variation in gene expression (GE) (Figure 4A); correspondence analysis (CA) was performed on the full dataset (Figure 4B). A principal component analysis (PCA) of the gene expression (GE) dataset revealed the presence of 5 clusters: (1) MoDCs, (2) ALDCs, (3) ALMacs, (4) MoMacs with alveolar Macs, and (5) blood monocytes. ALDCs and MoDCs had relatively similar GE profiles to each other which were significantly different to Macs with relatively similar GE profiles to each other (Figure 4A). In addition, the GE profiles of blood monocytes were distinctly different to all other cells analyzed. As for CA (Figure 4B), the list of the 20 genes responsible for the largest variations in inertia (quantity analogous to variance) is shown in Table 3. Noticeably, one single gene, SPP1, was responsible for more than the 10% of the total inertia.

**Table 3:** The 20 genes giving the largest contribution to inertia according to the Correspondence Analysis of Figure 4B.

**ENSEMBL gene name**	**Gene name**	**Inertia explained**	**Inertia explained (%)**
ENSBTAG00000005260	SPP1	0.058932831	10.2341
ENSBTAG00000011184	FTH1	0.018064914	3.13711
ENSBTAG00000043567		0.0154907	2.69008
ENSBTAG00000000604	GPNMB	0.014190275	2.46425
ENSBTAG00000009812		0.012897643	2.23977
ENSBTAG00000007622	CTSD	0.009037762	1.56948
ENSBTAG00000043570		0.008758801	1.52103
ENSBTAG00000015228	CD74	0.008169965	1.41878
ENSBTAG00000026779	LYZ	0.00775699	1.34706
ENSBTAG00000009359		0.006656633	1.15597
ENSBTAG00000007268	F13A1	0.006621972	1.14996
ENSBTAG00000021035	CTSK	0.00617882	1.073
ENSBTAG00000037811	CCL2	0.005152158	0.894711
ENSBTAG00000012442	CTSB	0.004747509	0.824441
ENSBTAG00000020676	MMP9	0.003996245	0.693978
ENSBTAG00000048122		0.003913428	0.679596
ENSBTAG00000026199	ACTB	0.003670953	0.637489
ENSBTAG00000047379		0.003585699	0.622684
ENSBTAG00000013472	COL1A2	0.003536053	0.614062
ENSBTAG00000032764		0.003415321	0.593096

*Inertia's role in Correspondence Analysis is similar to that played by variance in Principal Component Analysis*.

A grouped analysis of GE of all DCs and Macs showed that of almost 7,000 differentially expressed genes, over 5,000 were statistical and differentially expressed (*p* < 0.001, Figure 4C). For example, and confirming previous observations, transcription of CD14 and CD163 decreased in DCs (LogFC −3.03, *p* = 8.889 × 10^−23^ and LogFC −2.86, *p* = 1.324 × 10^−61^, respectively) whereas transcription of GM-CSFR (CSF3R) and CD1e increased in Macs (LogFC 2.76, *p* = 4.445 × 10^−149^ and LogFC 2.14, *p* = 2.362 × 10^−121^, respectively). Interestingly, transcription of CX3CR1, a marker typically used to define macrophage and DCs progenitors (MDP) (36) decreased in DCs (LogFC −2.52, *p* = 9.672 × 10^−23^). Other genes that could be identified as clear outliers in Figure 4C were CD4, CLMN, MYOF, ADAMTS14, SLC43A2, ADRB2, GALNT12, CARD14, VNN2, MS4A7, SLC6A12, and FABP4. Table 3 shows inertia analysis of highly statistically significant genes differentially expressed between subsets.

**Figure 5:**
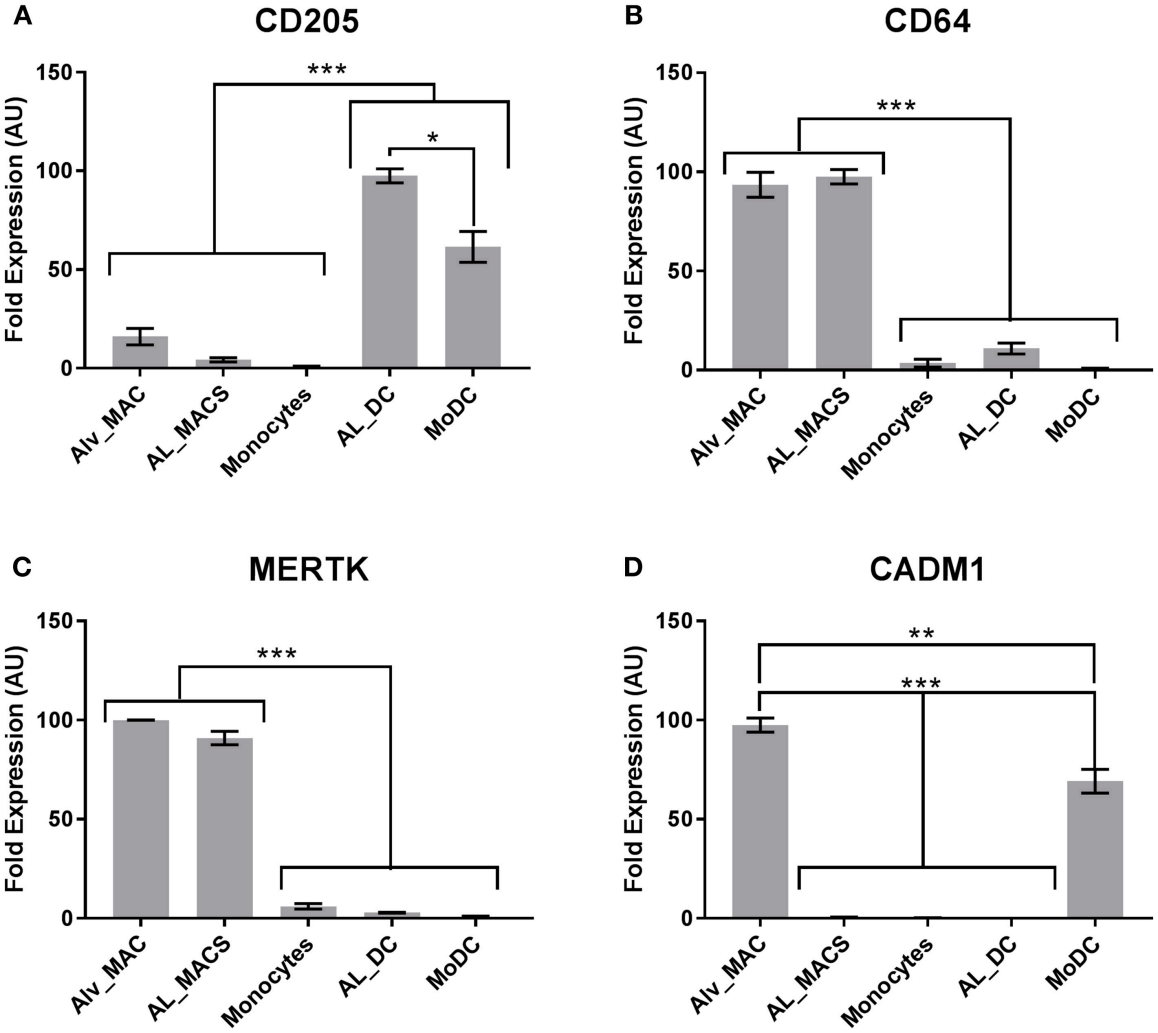
Bovine macrophages and DCs express common signatures with cells from other species. Bovine Macs and DCs (*N* = 4) were gated as in Figure 1 and sorted by flow cytometry. mRNA expression levels of **(A)** CD205, **(B)** CD64, **(C)** MERTK and **(D)** CADM1 were assessed by quantitative real-time PCR in triplicate. For each gene, data were normalized to the reference gene (18S rRNA) expression and presented as relative expression [arbitrary units (AUs)]: for each animal, the population with the highest expression for this gene was considered as 100 and the other populations were normalized to it. *p > 0.01, **p > 0.001, and ***p > 0.0001. Error bars indicate standard error of the means (SEM).

Next, a set of DE genes that were identified by high-throughput sequencing which have been suggested previously as markers to define mouse Macs and DCs (18) were validated by qRT-PCR. Unfortunately high quality RNA from MoMacs could not be obtained and so efforts were focused on the other cells available. Both MerTK and CD64 were confirmed to be highly transcribed in Macs but not DCs (Figures 5B,C) whereas CD205 was confirmed to be highly transcribed in DCs but not Macs (Figure 5A). CADM1 has been proposed to be highly expressed on DCs in pigs and mice (14) however our results showed it to be highly transcribed in macrophages and MoDCs but not in the other subsets (Figure 5D). These data indicate that mouse, human and bovine DCs and Macs have some common signatures that can be used to define functional similarities and differences but these signatures are not always universal.

## Discussion

As key mediators of T cell dependent immunity, DCs are considered primary targets for initiating immune responses. However, DCs can also play an important role in the induction of tolerance or autoimmunity. Whilst mouse and human DCs have been studied in detail, many of the highly specialized characteristics of DCs in other species remain poorly understood. Small numbers of DCs can be obtained as terminally differentiated, post-mitotic cells from either blood, spleen or other tissues or through the cannulation of lymphatic vessels. Good model cell lines therefore provide invaluable tools to study DCs biology; however these cell lines are generally not available for species other than humans and mice. Alternatively, DC-precursors, such as monocytes or bone marrow-derived cells, can be isolated and differentiated into DCs *in vitro*.

The bovine models of *in vitro*-derived DCs have been well established for many years and these models generally follow protocols designed to obtain human or mouse *in vitro*-derived DCs. Until recently, it was generally accepted that *in vitro*-derived DC cultures were composed of homogeneous populations of cells, since all cells were derived from common progenitors (CD14^+^ or bone marrow cells) and cells were cultured under the same conditions. A report by Helft et al. challenged that notion and clearly demonstrated that mouse *in vitro*-derived DCs were in fact a heterogeneous population of DCs and Macs (18, 37) which are derived from distinct but committed circulating precursors (38, 39).

In this work, we sought to define the populations of bovine *in vitro*-derived DC models and, like others, we have found cross-species similarities. *Bona fide* DCs can be obtained through cannulation of lymphatic vessels, a process that is not normally possible in humans or mice (19) or directly from blood (40); however blood DCs comprise a very small proportion of cells in the bovine system (41) and their use *in vitro* is unpractical. Bovine ALDCs have been shown to be FSC^hi^CD205^+^ and within this population, there are two distinct subpopulations defined by the differential expression of CD172a [SIRPα, (9, 19–21)]. Using the approach by Helft et al. (37), our results confirmed bovine ALDCs to be MHCII^++^CD11c^+^CD11b^−/+^CD205^+^CD1b^+/++^CD14^−^ CD172a^++/+^. As before, two distinct subpopulations were observed, CD172^++^ and CD172^+^. This phenotypic approach revealed the presence of bovine afferent-lymph macrophages (ALMacs) which had not been identified before and can now be defined as: MHCII^+^CD11c^+^CD11b^+^CD205^−^CD1b^+^CD14^−^CD172a^+^. Bovine ALDCs and ALMacs have distinct physical characteristics as observed by their FSC/SSC and by microscopy by resembling mouse and human DCs and Macs, respectively.

In the bovine system, the use of *in vitro* models of APC derived from blood monocytes is much more frequent than the use of BM-derived cells, mainly due to the ease of collection and volumes of peripheral blood available. Increased expression of CD1b has been the preferred method for determining the successful generation of bovine DCs from monocyte progenitors (7, 13). Importantly, the resulting MoDCs have almost universally been treated as homogeneous populations. We have studied monocyte-derived cultures in the context of heterogeneity, hypothesizing that these cultures could contain of a mixture of both DCs and Macs. CD1b expression increased in both MoMacs and MoDCs, which can only be used to define non-monocyte cells. Alternatively, only morphological changes have been used to demonstrate polarization of bovine CD14^+^ into Macs or DCs (34).

Adherent cells are generally thought to represent macrophages, however in our studies we found MoDC and MoMacs to be equally represented in both adherent and non-adherent monocyte-derived cells. Recently, Baquero and Plattner showed the use of CD11b, CD11c, CD163, CD205, CD14, and CD172a to differentiate bovine blood monocytes from *in vitro*-generated MoMacs and MoDCs (42), however all the antigens were used individually and the authors assumed that populations obtained in cell culture were homogeneous. However, our data proved otherwise: bovine MoDCs and MoMacs can be differentiated simply by plotting CD11c vs. MHCII surface expression and this separation can be confirmed by CD205 differential expression. We found that their phenotype closely resembles that of bovine ALDCs and ALMacs, respectively. In addition, our data showed that adherent and non-adherent MoDCs had identical phenotypes; the same applied for adherent and non-adherent MoMacs. We have also identified of SSC^high^ MHC^low^ CD14^int^ population and a separate SSC^low^ MHC^low^ CD14^high^ one in the afferent lymph that corresponded to granulocytes and monocytes, respectively. Bonneau et al. have previously identified these two populations in the afferent lymph of sheep and showed that these are major carriers of *Salmonella* from peripheral tissues to draining lymph nodes (32). Studies to identify functions of bovine afferent lymph granulocytes and monocytes are currently under way. We have previously shown that the frequency of ALDCs increases with inflammation [mechanical injury, infection of the skin, etc. (20)] but we have not yet investigated the effect of inflammation on ALMacs.

In our studies, the frequency of MoDCs and MoMacs obtained in cell cultures varied depending on the source of serum used in culture medium, length of culture, and immune status of the donor animals. In our hands, the presence or absence of Flt3L did not result in a preferential growth or “polarization” of DCs over Macs. This is not surprising as it has been shown in several studies that blood monocytes and monocyte-derived cells do not express Flt3 (17, 43). Similarly, the absence of Flt3L and/or IL4 did not result in a preferential growth or “polarization” of Macs over DCs. The ratio of MoDCs:MoMacs obtained was highly variable in between experiments even when using cells from the same donor and identical culture conditions (same source/batch of FCS, cytokines, culture medium, etc.), sometimes as low as 30% MoDCs:70% MoMacs and as high as 92% MoDCs:8% MoMacs. However, other culture conditions that may influence cells maturation were not tested and it was possible that specific growth conditions could result in preferential growth of either DCs or Macs. There is also evidence that recombinant proteins biological activity, type of medium used and even the source of the plastics used in culture plates have profound effects on monocyte maturation (44); however, these issues were not investigated in the current study. Hope and colleagues showed that the use of recombinant Flt3L alongside GM-CSF and IL4 helped in generation of more potent allo-stimulatory cells compared to cells grown in the absence of rFlt3L, suggesting that the use of these growth conditions contributed to DC generation (13). However, this fact remains controversial in light that monocyte-derived cells do not express Flt3. Undoubtedly, growth conditions impact *in vitro* maturation and ultimately biological function.

Co-stimulatory molecules expression has been proposed to be useful in distinguishing the various cells of the monophagocytic cell system, however differences in flow cytometric reagents, protocols, and instruments makes this process somewhat subjective. Therefore, the average number of surface antigens molecules per cell was determined using a standardized linear regression model. This approach confirmed that DCs had on average higher number of cell-surface co-stimulatory molecules compared to Macs and the corresponding monocytes. However, in this occasion we did not test the hypothesis that cellular activation increases the overall number of surface co-stimulatory molecules. Another parameter used to differentiate Macs from DCs was their phagocytic capacity (45). Here, we showed that MoDCs exhibited increased phagocytic activity compared with MoMacs, but both ALDCs and ALMacs had similar phagocytic capacities. In our studies, we did not separate MoDCs and MoMacs for phagocytosis assays. We observed that if in a particular culture there were more DCs than Macs, then the overall phagocytic activity of the whole culture was much higher than if there were more Macs than DCs, which reinforces the notion that the ratios of DC:Macs influence the outcome and therefore conclusions drawn from the experiments in question.

A global transcript analysis revealed that there were numerous differentially expressed genes between the two populations. As expected, a large number of transcripts related to the complement pathway were expressed in Macs and not in DCs, as well as the previously-defined transcription factor MerTK. Therefore, MerTK can be used to differentiate between Macs and DCs across different species. Osteopontin (encoded by SSP1) functionally activates dendritic cells (46) and promotes inflammatory responses (47, 48). Interestingly, osteopontin (encoded by SSP1) functionally activates dendritic cells (46) and promotes inflammatory responses (47, 48). Whether the transcription of SPP1 and subsequent expression of osteopontin by various subsets of bovine monophagocytic cells delineates “pro-inflammatory” and “anti-inflammatory” cells as proposed for human cells (49) is certainly worth investigating. However, results generated in mice are contradictory (50) so it is possible that there are species-specific differences. Interestingly, a large number of interferon regulatory factor (IRF) transcripts were found to be preferentially expressed in bovine DCs compared to Macs, suggesting that genes regulated by the interferon pathway were highly expressed in DCs compared to Macs.

Molecular tools are now used to help define cell subsets. High throughput sequencing reveals clear differences between putative Macs and DCs populations. Our data support the notion that a conserved set of genes can be used to differentiate and identify Macs from DCs, in all species and in cells from different sources. For example, the transcription factor MerTK was proposed as a definitive marker of Macs in mice and this appears to be the true in cattle and pigs alike. On the other hand, CADM1 has been proposed to be solely expressed on DCs and not on Macs (14, 51); in contrast to data published for pigs, we found transcription of CADM1 on bovine alveolar macrophages and absence of transcription on ALDCs. It is possible that bovine cells have evolved in such a way that CADM1 cannot be used to differentiate between DCs and Macs or that the experimental conditions were such that we did not detect any differential transcription in the cells in question. Alternatively, differential expression of complement-related genes could also be useful, as there was a clear increased transcription of complement-related genes in Macs. A better understanding of conserved gene expression across different species will increase our knowledge of how different cells interact with pathogens across species.

On the other hand, it has been proposed that DCs as such are only a heterogeneous subset of mononuclear phagocytes, part of a diverse and plastic mononuclear phagocytic cell system (52). How DCs and Macs are defined may seem relative and trivial; however given the various *in vitro* systems, growth conditions used and conclusions made based on biological activity, these definitions are clearly important. Growth conditions and phenotypic definitions need to be standardized across the various laboratories in order to make comparable studies and biologically valid conclusions. Most *in vitro* systems rely on the maturation of cells in the presence of recombinant cytokines but some studies do not follow these protocols. For example, Casey and colleagues (53) analyzed global gene expression in bovine MoMacs infected with *Mycobacterium bovis*, but their culture conditions did not include recombinant cytokines, therefore direct comparison with other studies cannot be made.

As mentioned before, several studies have tried to identify signatures associated with the various components of the MPC system not only in single species but across species as well. We have tried to aggregate the most important phenotypic and transcriptomic features helping to define the various members of the MPC system and which are consistent across species (Table 4). In the particular case of the bovine system, two recent studies have used large panels of antibodies to try and define bovine MPC. Our results agree with Park et al (16) in that both MoDCs and MoMacs express MHCII, CD1b, CD11c, CD14, and CD172; however, in our hands, MoMacs did not express CD205 contrary to their report showing that this antigen is indeed expressed on MoMacs. Experimental conditions could be the reason behind this discrepancy. Talker et al. (17) did not look at monocyte-derived cells, but showed that bovine cDC present in peripheral blood also express MHCII, CD11c, and CD205. These results for bovine DCs are in agreement with those of human MoDCs (57), human lymph node-derived cells (58) and it is confirmed by transcriptional analysis of DCs across various species (43). In conclusion, the triple analysis of MHCII, CD11c, and CD205 can be used effectively to identify and differentiate DCs and Macs across different species.

**Table 4:** Summary of marker combinations for identifying mononuclear phagocytic cells across species (humans, mice, cattle, and pigs) based on phenotypic and transcriptomic data.

	**Blood Monocytes (2, 8, 32, 39, 43, 49, 54–56)**	**cDC (5, 8, 16, 17, 39, 43, 44, 51, 54, 57–59)**	ALDC ^*^ (9, 15, 19–21, 32, 48, 60, 61)	ALMacs ^*^ (15, 60)	**MoDC (1, 3–8, 12–14, 16, 18, 34, 37, 38, 42–44, 54, 61, 62)**	**MoMacs (5, 6, 10–12, 16, 18, 34–37, 39, 43, 49, 51, 52, 58, 63)**	**Lung Macs (26, 63–65)**
MHCII	+	++	++	+	++	+	+
CD11c	+	+	+	+	+	+	+
CD1b	–	+	+	+	+	+	+
CD14	+(1)	–	–	–	+(2)	+	+
CD172a	+	– or + (3)	++/+ (4)	+	–/+ (5)	– or + (7)	+
CD205	–	+	+	–	+	–	–
CD209	–	+	+ (6)	–	+	+	+
Flt3	–	–	+	–	–	–	+
CD163	– or + (7)	– or + (7, 8)	–	+	– or + (7, 8)	+	+
CX3CR1	–	+ or – (9)	+	–	+ or – (9)	+	+
MerTK	–	–	–	+	–	+	+
CadM1	– or + (7)	– or + (7)	– or + (7)	– or + (7)	– or + (7)	– or + (7)	– or + (7)

* *Afferent lymph cells (10, 17) in cattle and pigs; lymph node derived cells in humans*.

*(1) A minor subset is CD14 negative*.

*(2) Human MoDCs loose CD14 expression*.

*(3) Cattle and pigs have differential expression of CD172*.

*(4) Subpopulations of cells have differential expression of CD172*.

*(5) Conflicting data, this report and Vu Mahn et al. (43)*.

*(6) Subset specific. Negative expression of CD209 in human DCs Granelli-Piperno et al. (58)*.

*(7) Species-specific differences*.

*(8) Conflicting data, Park et al. (8) and Seo et al. (16)*.

*(9) Conflicting information, this report and Maisonnasse et al. (31) and Fogg et al. (36)*.

Expression of CD163 has also been proposed to be useful in identifying DCs: Park et al. (16) demonstrated that both bovine MoDCs and MoMacs expressed CD163, however Talker et al. (17) showed that bovine cDC do not express CD163 whereas monocytes do. Autenrieth et al. (57) also demonstrated that human MoDCs do not express CD163 whereas Maisonnasse et al. (31) indicated that pig alveolar Macs do express CD163 but various lung DCs can be identified by their differential expression of CD163. In our studies, the staining with anti-CD163 was inconsistent across experiments and samples; however, our transcriptomic analysis revealed that in all DCs studied the transcription of CD163 decreased compared to its transcription in Macs. Perhaps there were species-specific differences, CD163 expression could be tissue-specific or it could be dependent on the cells activation status at the time of analysis. CD163 expression and its relevance across species require further investigation.

One additional surface antigen used to identify MPC was DC-SIGN (CD209). In our studies, CD209 expression was not significantly different in blood monocytes, MoDCs or MoMacs; however, its expression increased in ALMacs and ALDCs compared with ALMonocytes. Our results were in contrast with those presented by Park et al. (16) where it was shown that both MoDCs and MoMacs expressed CD209. Previously, several studies have shown that cultures of human blood monocytes with IL4 result in CD209 rapid expression (57, 58) so it is plausible to think that our experimental conditions did not result in the detection of CD209 by flow cytometry at the point of highest expression. Certainly, this phenomenon needs to be investigated further.

The ultimate test to differentiate between DCs and Macs is their differential capacity to present antigen and activate T cells. These antigen presentation studies also help differentiate between DC subsets and Macs subsets. In this study, we relied in two proxy measures of antigen presentation efficacy: (1) expression of co-stimulating antigens and (2) phagocytic capacity. Antigen presentation functional studies that will help confirm the identity of these cells are being carried out.

Vu Manh et al. (43) have reported a very complete comparative transcriptomic analysis of MPC across human, mouse, sheep and pigs. In our studies we did not differentiate between the various DC subsets (cDC1, cDC2, pDC), Mac subsets (Mac1, Mac2) or monocyte subsets (classical, intermediate, non-classical); however we have generated a large mRNA transcriptomic dataset of bovine MPC and it would be very useful to include our data to increase our knowledge of the ontogeny, evolution and function of MPC across different species.

In conclusion, we have evaluated bovine Macs and DC phenotypically and genetically. We showed that monocyte-derived *in vitro* cultures were made up by heterologous populations of cells with distinct biological activity, phenotypic and molecular signatures. Also, the ratio of DCs to Macs was variable in our cultures and it was dependent on many conditions such as health status of the donor animal, type of plastic used, length of culture conditions, etc. Bovine afferent-lymph macrophages were defined for the first time and our results showed that these cells have a distinct biological activity, phenotypic and molecular signatures. Our data support the notion that traditional approaches to define mononuclear phagocyte populations based on phenotype only require to be revised and must take into account origin, gene expression patterns and experimental conditions. It is important to evaluate each subset status in the mixed population used for *in vitro* studies when comparing and reproducing experiments, which will also allow us better understanding in the implication of each subset. In addition more work is required to resolve phenotypic differences observed by different investigators and across species and their relevance in APC function. Refinement of *in vitro* DCs culture systems will inevitably lead to a better understanding of DCs and Macs function which has important implications for the design of vaccines and immune responses.

## Author Contributions

EG and MM: carried out experimental design and co-wrote the manuscript. EG: carried out the studies on bovine cells. MP: carried out gene expression analysis. PR: analyzed the transcriptomic data.

### Conflict of Interest Statement

The authors declare that the research was conducted in the absence of any commercial or financial relationships that could be construed as a potential conflict of interest.
