# Supplementary material for: Bovine Derived in vitro Cultures Generate Heterogeneous Populations of Antigen Presenting Cells
Source: Front Immunol. 2019 Mar 29;10:612. doi: 10.3389/fimmu.2019.00612 (PMC6450137; doi:10.3389/fimmu.2019.00612)
Supplement: Supplementary file 1 [file Data_Sheet_1.PDF]

Supplementary Figure 1

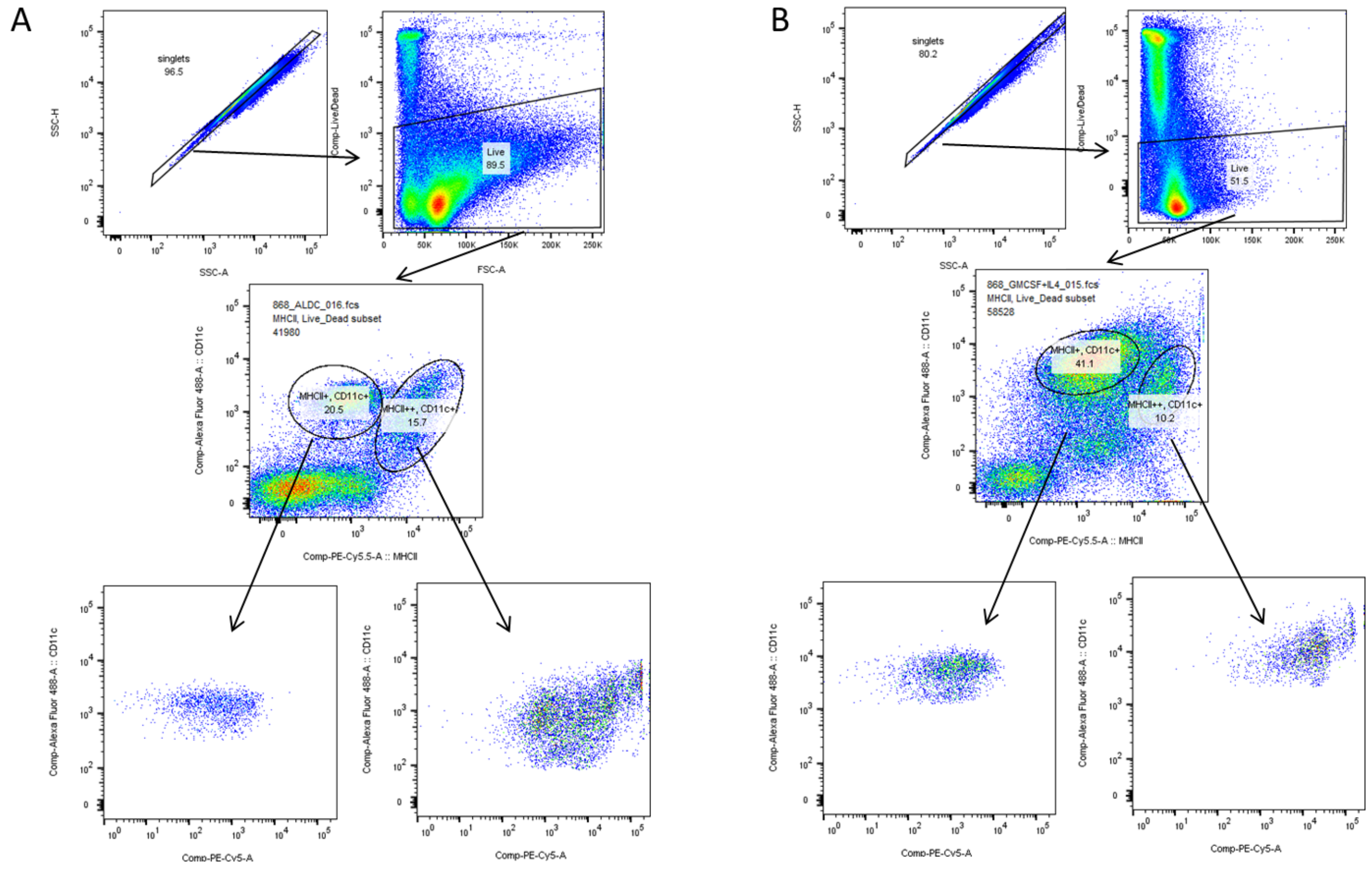

S1. Flow sorting of subpopulations of monophagocytic cells. Singlets-Live events of A) afferent lymph and B) monocyte-derived cells were stained for surface expression of MHCII and CD11c and the main populations identified by the gates flow sorted using a FACSAria III. Aliquots of the sorted populations were re-analyzed to confirm the phenotype of the sorted populations. Representative samples of cells from 6 different animals
