# Supplementary material for: Bovine Derived in vitro Cultures Generate Heterogeneous Populations of Antigen Presenting Cells
Source: Front Immunol. 2019 Mar 29;10:612. doi: 10.3389/fimmu.2019.00612 (PMC6450137; doi:10.3389/fimmu.2019.00612)
Supplement: Supplementary file 2 [file Data_Sheet_2.PDF]

Supplementary Figure 2

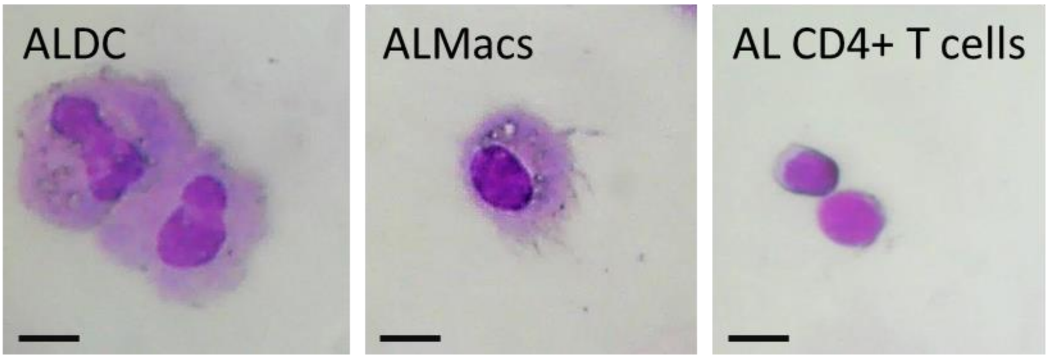

S2. Giemsa-stained cytospins of flow-sorted ALDCs ($MHCII^{++}CD11c^{+}CD205^{+}$), AlMacs ($MHCII^{+}CD11c^{+}CD205^{-}$), and AL $CD4^{+}$ T cells ($MHCII^{-}CD11c^{-}CD205^{-}CD3^{+}CD4^{+}$). Black bar represents 10 µm.
